# Supplementary material for: An analysis of the 24-hour on-call experience and treatment decision of a dental resident, a retrospective study
Source: PeerJ. 2025 Jan 30;13:e18678. doi: 10.7717/peerj.18678 (PMC11787798; doi:10.7717/peerj.18678)
Supplement: Supplemental Information 2 [file peerj-13-18678-s002.docx]

Sex

1, male; 2, female

Normal or extending time

1, normal time; 2, extending time

Dental interventions

1, yes; 2, no

因变量

dependent variable

Major

1, Endodontics; 2, periodontics; 3, Prosthodontics;4, Oral and Maxillofacial Surgery;5, Pediatric Dentistry; 6. Implant Dentistry; 7. Orthodontics

预期有差异

Meaningful independent variables

Level

PG4, postgraduate year 4; PG5, postgraduate year 5; PG6, postgraduate year 6;

预期有差异

Meaningful independent variables
